# Teaching and Safety-Net Hospital Penalization in the Hospital-Acquired Condition Reduction Program

**DOI:** 10.1001/jamanetworkopen.2023.56196

**Published:** 2024-02-16

**Authors:** Jose A. Serpa, Gretchen Gemeinhardt, Cesar A. Arias, Robert O. Morgan, Heidi Russell, Hongyu Miao, Cecilia M. Ganduglia Cazaban

**Affiliations:** 1 Section of Infectious Diseases, Department of Medicine, Baylor College of Medicine, Houston, Texas; 2 Department of Management, Policy and Community Health, University of Texas School of Public Health, Houston; 3 Center for Infectious Diseases, Division of Infectious Diseases, Department of Medicine, Houston Methodist Hospital, Houston Methodist Research Institute, Houston, Texas; 4 Department of Medicine, Weill Cornell Medical College, New York, New York; 5 Department of Statistics, and College of Nursing, Florida State University, Tallahassee

## Abstract

**Importance:**

The Hospital-Acquired Condition Reduction Program (HACRP) evaluates acute care hospitals on the occurrence of patient safety events and health care–associated infections. Since its implementation, several studies have raised concerns about the overpenalization of teaching and safety-net hospitals, and although several changes in the program’s methodology have been applied in the last few years, whether these changes reversed the overpenalization of teaching and safety-net hospitals is unknown.

**Objective:**

To determine hospital characteristics associated with HACRP penalization and penalization reversal.

**Design, Setting, and Participants:**

This retrospective cross-sectional study assessed data from 3117 acute care hospitals participating in the HACRP. The HACRP penalization and hospital characteristics were obtained from Hospital Compare (2020 and 2021), the Inpatient Prospective Payment System impact file (2020), and the American Hospital Association annual survey (2018).

**Exposures:**

Hospital characteristics, including safety-net status and teaching intensity (no teaching and very minor, minor, major, and very major teaching levels).

**Main Outcomes and Measures:**

The primary outcome was HACRP penalization (ie, hospitals that fell within the worst quartile of the program’s performance). Multivariable models initially included all covariates, and then backward stepwise variable selection was used.

**Results:**

Of 3117 hospitals that participated in HACRP in 2020, 779 (25.0%) were safety-net hospitals and 1090 (35.0%) were teaching institutions. In total, 771 hospitals (24.7%) were penalized. The HACRP penalization was associated with safety-net status (odds ratio [OR], 1.41 [95% CI, 1.16-1.71]) and very major teaching intensity (OR, 1.94 [95% CI, 1.15-3.28]). In addition, non–federal government hospitals were more likely to be penalized than for-profit hospitals (OR, 1.62 [95% CI, 1.23-2.14]), as were level I trauma centers (OR, 2.05 [95% CI, 1.43-2.96]) and hospitals located in the New England region (OR, 1.65 [95% CI, 1.12-2.43]). Safety-net hospitals with major teaching levels were twice as likely to be penalized as non–safety-net nonteaching hospitals (OR, 2.15 [95% CI, 1.14-4.03]). Furthermore, safety-net hospitals penalized in 2020 were less likely (OR, 0.64 [95% CI, 0.43-0.96]) to revert their HACRP penalization status in 2021.

**Conclusions and Relevance:**

Findings from this cross-sectional study indicated that teaching and safety-net hospital status continued to be associated with overpenalization in the HACRP despite recent changes in its methodology. Most of these hospitals were also less likely to revert their penalization status. A reevaluation of the program methodology is needed to avoid depleting resources of hospitals caring for underserved populations.

## Key Points

**Question:**

Do teaching and safety-net hospitals continue to be overpenalized by the Hospital-Acquired Condition Reduction Program (HACRP) after the introduction of recent methodology changes in the program?

**Findings:**

In this cross-sectional study of 3117 acute care hospitals, teaching and safety-net hospitals continued to be more frequently penalized in recent HACRP versions. Safety-net hospitals with major teaching levels were twice as likely to be penalized as non–safety-net nonteaching hospitals.

**Meaning:**

Study findings suggest that reevaluation of the program methodology is needed to avoid depleting the resources of hospitals caring for underserved populations.

## Teaser

This cross-sectional study assesses hospital characteristics associated with Hospital-Acquired Condition Reduction Program penalization and penalization reversal after the introduction of recent program methodology changes among acute care hospitals.

## Introduction

The Hospital-Acquired Condition Reduction Program (HACRP) is a pay-for-performance program that was established by the Patient Protection and Affordable Care Act in 2010 and was first implemented by the Centers for Medicare & Medicaid Services (CMS) in fiscal year (FY) 2015.^1^ The HACRP applies to all acute care hospitals participating in the inpatient prospective payment system, except for hospitals in the state of Maryland, which are on an all-payer system under a Medicare waiver.^2^ In the HACRP, each hospital is assigned a total performance score, calculated as the average of the patient safety indicator composite (PSI) score and 5 health care-associated infection (HAI) scores.^3^ The PSI score is calculated using administrative claims data.^4^ The HAI scores are reported by the National Healthcare Safety Network after reviewing hospital records and laboratory results.^5^ For FY 2020 and 2021, HAIs evaluated by the HACRP included central line–associated bloodstream infection, catheter-associated urinary tract infection, surgical site infection (abdominal hysterectomy and colon procedures), methicillin-resistant *Staphylococcus aureus* bacteremia, and *Clostridioides difficile* infections.^3^

The HACRP does not evaluate clinical outcomes such as mortality or readmissions; however, the program assesses hospital patient safety events and HAIs, which are 2 important causes of mortality and morbidity.^6^ Hospitals whose HACRP total performance scores rank in the worst quartile are subjected to a 1% payment reduction in their overall Medicare fee-for-service discharge payments.^1^ Best-performing hospitals do not receive any financial incentives. Some studies evaluating the performance of the HACRP prior to 2018 have raised concerns about the validity and reliability of the HACRP methodology^7,8,9,10,11,12^ as well as its marginal association with reduced rates of hospital-acquired conditions.^13,14,15^ The HACRP performance scores also correlated poorly with surveys of patient satisfaction.^16^ Most importantly, in those earlier versions of the program, the HACRP seemed to overpenalize hospitals with certain structural characteristics. For instance, large teaching and safety-net hospitals and hospitals offering more complex services and having more specialty accreditations were more likely to be penalized than small private hospitals.^13,17,18,19,20^

The CMS implemented several changes in the HACRP methodology in 2018 and 2019 to tackle those drawbacks. Those modifications involved the use of winsorized scores to report hospitals’ individual and total performance scores,^21^ the adoption of a recalibrated version (using updated risk and reliability adjustment models as well as composite weights) of the PSI composite score,^21^ and the substitution of a weighted scoring system for an equal average of all available scores reported by a hospital.^3^

Whether large teaching and safety-net hospitals continue to be overpenalized by the HACRP after the introduction of those recent program methodology changes is unknown. Using recent versions of the HACRP (FY 2020 and 2021), we conducted a study to identify hospital structure characteristics associated with HACRP penalization and penalization reversal.

## Methods

Because this cross-sectional study was non–human participants research, it was considered exempt from review and the requirement to obtain informed consent was waived by the University of Texas Health Science Center at Houston Committee for the Protection of Human Subjects. We performed a retrospective cross-sectional study, using secondary data on all acute care hospitals participating in the HACRP for FY 2020 and 2021 (3224 and 3204 hospitals, respectively). This report follows the Strengthening the Reporting of Observational Studies in Epidemiology (STROBE) reporting guideline.

Study data were obtained from databases available on the Hospital Compare website, including for HACRP FY 2020 and 2021.^22^ For each hospital participating in the HACRP, we recorded penalization status for FY 2020 and FY 2021 and the HACRP total performance scores. The eFigure in Supplement 1 shows a visual representation of the hospitalization timeline contained within each database. Due to the COVID-19 pandemic, hospitals were exempted from mandatory reporting of HAI data for quarter 4 of 2019 (October 1 through December 31, 2019). Reporting for that quarter was voluntary.^1^

We also collected data on hospital size, region, intensity of resident teaching activities, case mix index, and disproportionate share hospital patient (DSHP) percentages from the Inpatient Prospective Payment System (impact file) FY 2020.^23^ Teaching intensity was defined using the resident to bed ratio variable. As in previous studies,^17,24^ hospitals were categorized by 5 teaching intensity groups, including no teaching (resident to bed ratio, 0), very minor teaching (resident to bed ratio, 0.001-0.049), minor teaching (resident to bed ratio, 0.050-0.249), major teaching (resident to bed ratio, 0.250-0.599), and very major teaching (resident to bed ratio, ≥0.600). Safety-net hospital status was defined as hospitals in the highest quartile of the DSHP percentage.^17,25^

Data on organizational structure characteristics, including hospital type, admissions, accreditations, transplant services, level I trauma designation, and nurse to bed ratios were obtained from the American Hospital Association (AHA) annual survey. The AHA survey collects data on organizational structure, facilities and services, insurance and alternative payment models, total facility beds, finances, and staffing from over 6000 hospitals and 400 health care systems in the United States.^26^ We selected the 2018 annual survey as it contained the hospitalization periods for the studied HACRP years.

### Statistical Analysis

#### HACRP Penalization and Hospital Characteristics

Conventional measurements of central location and dispersion were used to describe the data. Hospital structure characteristics were compared according to HACRP penalization status and tested for significant differences by using χ^2^ tests or Fisher exact tests for categorical variables and *t* tests or Mann-Whitney tests for continuous variables. For variable selection in our model, we started with a model that included all covariates and then used backward stepwise variable selection. We also fitted a model to determine hospital characteristics associated with HACRP penalization by including a preselected interaction between teaching level and safety-net status. Furthermore, we used the least absolute shrinkage and selection operator (LASSO) for inference to determine the effect size (ie, the strength of the association) for our 2 main variables: teaching level and safety-net status. Pairwise interactions for all other covariates were included in LASSO inference commands. We performed several sensitivity analyses. We used alternative definitions for teaching hospitals (based on their membership in the Council of Teaching Hospitals and Health Systems [COTH] or having major or very major teaching intensity) and for safety-net hospitals (defined as hospitals in the highest tertile of the DSHP). In another sensitivity analysis, we included the variable resident to bed ratio as a continuous variable by using restricted cubic splines in a backward stepwise model. In addition, we compared our results with previously described hospital characteristics associated with HACRP penalization in FY 2015. In addition, we compared our results with previously described hospital characteristics associated with HACRP penalization in FY 2015.^17^

We also conducted a separate analysis to evaluate associations between the HACRP total performance scores (continuous variable) and hospital characteristics. Because the dependent variable (HACRP total performance scores) was found to be nonnormally distributed (by the Shapiro-Wilk test) and had outliers, we used nonparametric bivariate analyses and conducted quantile regression analyses to calculate coefficient estimates of covariates associated with HACRP scores at the 25th (best-performing hospitals), 50th (median-performing hospitals), and 75th (worst-performing hospitals) quantiles.

#### HACRP Penalization Reversal and Hospital Characteristics

We determined hospital characteristics associated with the reversal of the HACRP penalization by comparing the characteristics of hospitals penalized in FY 2020 but not penalized in FY 2021 with those that remained penalized in FY 2021. For variable selection in our model, we used backward stepwise variable selection. We also used LASSO for inference to determine the effect size of the associations with teaching levels and safety-net status.

Statistical significance was defined as a 2-sided *P* < .05. All statistical analyses were performed in Stata, version 17 SE (StataCorp LLC).

## Results

### HACRP Penalization and Hospital Characteristics

The HACRP FY 2020 database (3224 hospitals) was merged with the impact file and the AHA survey by using the facility (hospital) identification number as the key matching variable. This resulted in 3163 matched hospitals (and 61 unmatched hospitals) (Figure). Unmatched hospitals were manually checked to confirm that they lacked relevant data. Among unmatched hospitals, 29 (47.6%) had some data for review. They were more likely than matched hospitals to be small hospitals and nonteaching institutions. Unmatched and matched hospitals had similar proportions of safety-net institutions. Penalization status was not provided for 46 Maryland hospitals, so they were excluded, resulting in 3117 hospitals participating in HACRP FY 2020. Of those hospitals, 779 (25.0%) were safety-net hospitals and 1090 (35.0%) were teaching institutions. Among the teaching hospitals, 305 (28.0%) had very minor teaching intensity levels, 467 (42.8%) minor teaching intensity levels, 232 (21.3%) major teaching intensity levels, and 86 (7.9%) very major teaching intensity levels. The median (IQR) annual number of hospital admissions was 6679 (2456-13 791), and the median (IQR) case mix index was 1.62 (1.41-1.83). The median (IQR) nurse to bed ratio was 1.49 (1.08-1.97), and the median (IQR) inpatient surgical procedure to bed ratio was 10.53 (7.10-14.65) (Table 1). In total, 771 hospitals (24.7%) were penalized by the HACRP in FY 2020. Penalized hospitals were more likely to be safety-net hospitals, be larger, have more admissions, be accredited by the Commission on Cancer, offer transplant services, be a level I trauma center, have a higher nurse to bed ratio, be in the New England region compared with other regions of the country, and be non–federal government compared with investor-owned for-profit hospitals (Table 1).

**Figure.**
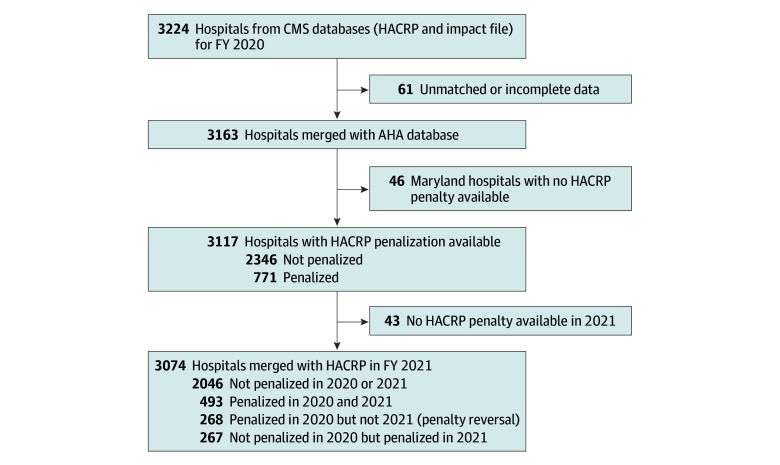
Study Flowchart AHA indicates American Hospital Association; CMS, Centers for Medicare & Medicaid Services; FY, fiscal year; and HACRP, Hospital-Acquired Condition Reduction Program.

**Table 1.** Hospital Structure Characteristics by Penalization Status in HACRP Fiscal Year 2020

Characteristic	Hospitals, No. (%)			*P* value^a^
	Penalized	Not penalized	Total	
No. of hospitals	771 (24.7)	2346 (75.3)	3117 (100)	NA
Resident to bed ratio, median (IQR)	0 (0-0.136)	0 (0-0.034)	0 (0-0.052)	<.001
Resident to bed ratio, categories				
No teaching (0)	439 (56.9)	1588 (67.7)	2027 (65.0)	<.001
Very minor teaching (0.001-0.049)	77 (10.0)	228 (9.7)	305 (9.8)	
Minor teaching (0.050-0.249)	122 (15.8)	345 (14.7)	467 (15.0)	
Major teaching (0.250-0.599)	85 (11.0)	147 (6.3)	232 (7.4)	
Very major teaching (≥0.600)	48 (6.2)	38 (1.6)	86 (2.8)	
DSHP				
Quartile 1 (≤0.19)	148 (19.2)	633 (27.0)	781 (25.1)	<.001
Quartile 2 (0.20-0.28)	178 (23.1)	603 (25.7)	781 (25.1)	
Quartile 3 (0.29-0.38)	190 (24.6)	586 (25.0)	776 (24.9)	
Quartile 4 (>0.38)	255 (33.1)	524 (22.3)	779 (24.9)	
Safety-net hospital^b^				
No	516 (66.9)	1822 (77.7)	2338 (75.0)	<.001
Yes	255 (33.1)	524 (22.3)	779 (25.0)	
Hospital bed size category				
Small (<100 beds)	217 (28.1)	843 (35.9)	1060 (34.0)	<.001
Medium (100-399 beds)	396 (51.4)	1215 (51.8)	1611 (51.7)	
Large (≥400 beds)	158 (20.5)	288 (12.3)	446 (14.3)	
Total hospital admissions				
Quartile 1 (≤2456)	160 (20.8)	620 (26.4)	780 (25.0%)	<.001
Quartile 2 (2457-6679)	175 (22.7)	604 (25.8)	779 (25.0%)	
Quartile 3 (6680-13 791)	208 (27.0)	571 (24.3)	779 (25.0%)	
Quartile 4 (>13 791)	228 (29.5)	551 (23.5)	779 (25.0%)	
Case mix index				
Quartile 1 (≤1.41)	172 (22.3)	608 (25.9)	780 (25.0%)	.17
Quartile 2 (1.42-1.62)	190 (24.7)	589 (25.1)	779 (25.0%)	
Quartile 3 (1.63-1.83)	203 (26.3)	576 (24.6)	779 (25.0%)	
Quartile 4 (>1.83)	206 (26.7)	573 (24.4)	779 (25.0%)	
Accredited by Joint Commission				
No	177 (23.0)	558 (23.8)	735 (23.6)	.64
Yes	594 (77.0)	1788 (72.2)	2382 (76.4)	
Accredited by DNV				
No	705 (91.4)	2099 (89.5)	2804 (90.0)	.12
Yes	66 (8.6)	247 (10.5)	313 (10.0)	
Commission on Cancer accreditation				
No	415 (53.8)	1426 (60.8)	1841 (59.0)	.001
Yes	356 (46.2)	920 (39.2)	1276 (41.0)	
Council of Teaching Hospitals				
No	664 (86.1)	2220 (94.6)	2884 (92.5)	<.001
Yes	107 (13.9)	126 (5.4)	233 (7.5)	
Transplant services				
No	495 (64.2)	1687 (71.9)	2182 (70.0)	<.001
Yes	276 (35.8)	659 (28.1)	935 (30.0)	
Level I trauma center				
No	655 (84.9)	2224 (94.8)	2879 (92.4)	<.001
Yes	116 (15.1)	122 (5.2)	238 (7.6)	
Nurse to bed ratio				
Quartile 1 (≤1.08)	185 (24.0)	595 (25.4)	780 (25.0%)	.01
Quartile 2 (1.09-1.49)	175 (22.7)	604 (25.7)	779 (25.0%)	
Quartile 3 (1.50-1.97)	184 (23.9)	596 (25.4)	780 (25.0%)	
Quartile 4 (>1.97)	227 (29.4)	551 (23.5)	778 (25.0%)	
Inpatient surgical procedures per bed ratio				
Quartile 1 (≤7.10)	194 (25.2)	586 (25.0)	780 (25.0%)	.92
Quartile 2 (7.11-10.53)	188 (24.4)	591 (25.2)	779 (25.0%)	
Quartile 3 (10.54-14.65)	199 (25.8)	580 (25.8)	779 (25.0%)	
Quartile 4 (>14.65)	190 (24.6)	589 (24.6)	779 (25.0%)	
Type of hospital control				
Investor owned, for profit	148 (19.2)	578 (24.6)	726 (23.3)	<.001
Nongovernment, not for profit	474 (61.5)	1472 (62.8)	1946 (62.4)	
Government, nonfederal	149 (19.3)	296 (12.6)	445 (14.3)	
Hospital region				
New England	45 (5.8)	86 (3.7)	131 (4.2)	.046
Mid Atlantic	95 (12.3)	256 (10.9)	351 (11.3)	
South Atlantic	126 (16.4)	384 (16.4)	510 (16.4)	
East North Central	114 (14.8)	369 (15.7)	483 (15.5)	
East South Central	66 (8.6)	219 (9.3)	285 (9.1)	
West North Central	61 (7.9)	188 (8.0)	249 (8.0)	
West South Central	101 (13.1)	389 (16.6)	490 (15.7)	
Mountain	52 (6.7)	174 (7.4)	226 (7.3)	
Pacific	111 (14.4)	281 (12.0)	392 (12.5)	

Abbreviations: DNV, Det Norske Veritas; DSHP, disproportionate share hospital patient percentage; HACRP, Hospital-Acquired Condition Reduction Program; NA, not applicable.

^a^ *P* values are for the comparison between penalized and nonpenalized hospitals.

^b^ Safety-net hospitals were defined as those in the highest quartile of the DSHP.

Hospitals with higher teaching intensity were also more likely to be penalized than nonteaching hospitals. For example, 55.8% and 36.6% of very major and major teaching hospitals were penalized compared with 21.7% of nonteaching hospitals. Hospitals that were members of COTH (233 of 1090 [21.4%] teaching hospitals) were also more likely to be penalized than nonmember hospitals (45.9% vs 23.0%). Accreditation by the Joint Commission or Det Norske Veritas (DNV) was not associated with penalization (Table 1).

When we compared our results with previously published HACRP data from FY 2015,^17^ we noticed an increase in the penalization rates of small hospitals (21.8% in 2015 vs 28.1% in 2020) with a corresponding decrease in the rates of penalization of medium (54.4% in 2015 vs 51.4% in 2020) and larger (23.8% in 2015 vs 20.5% in 2020) hospitals (*P* = .01). Similarly, we observed a decrease in the penalization of hospitals in the highest quartiles of hospital admissions (39.7% in 2015 vs 29.6% in 2020) and case mix indexes (37.3% in 2015 and 26.7% in 2020) (both *P* < .001). There was no significant change in the proportions of safety-net and teaching hospitals penalized in FY 2015 and FY 2020.

In our multivariable model, HACRP penalization was associated with safety-net status (odds ratio [OR], 1.41 [95% CI, 1.16-1.71), very major teaching intensity (OR, 1.94 [95% CI, 1.15-3.28]), non–federal government hospitals (OR, 1.62 [95% CI, 1.23-2.14]), and level I trauma center status vs nontrauma centers (OR, 2.05 [95% CI, 1.43-2.96]). Hospitals in the New England region were also more frequently penalized than hospitals in other regions of the country (OR, 1.65 [95% CI, 1.12-2.43]) (Table 2).

**Table 2.** Hospital Structure Characteristics Associated With Hospital-Acquired Condition Reduction Program Penalty Status (Categorical Outcome)

Characteristic	Backward elimination model, OR (95% CI)	Model with interaction term, OR (95% CI)	LASSO inference for teaching and safety-net hospitals, OR (95% CI)
Resident to bed ratio categories			
No teaching	1 [Reference]	1 [Reference]	1 [Reference]
Very minor teaching	1.14 (0.84-1.55)	1.16 (0.81-1.64)	1.16 (0.86-1.58)
Minor teaching	1.08 (0.83-1.41)	1.17 (0.86-1.58)	1.08 (0.82-1.42)
Major teaching	1.27 (0.89-1.81)	0.90 (0.57-1.43)	1.25 (0.86-1.83)
Very major teaching	1.94 (1.15-3.28)	1.30 (0.50-3.40)	1.93 (1.13-3.29)
Safety-net hospital			
No	1 [Reference]	1 [Reference]	1 [Reference]
Yes	1.41 (1.16-1.71)	1.32 (1.03-1.72)	1.34 (1.09-1.65)
Teaching × safety-net interaction			
No teaching, no safety-net	NA	1 [Reference]	NA
Very minor teaching, safety-net	NA	0.96 (0.51-1.83)	NA
Minor teaching, safety-net	NA	0.79 (0.47-1.34)	NA
Major teaching, safety-net	NA	2.15 (1.14-4.03)	NA
Very major teaching, safety-net	NA	1.81 (0.62-5.33)	NA
Level I trauma center, safety-net			
No	1 [Reference]	1 [Reference]	NA
Yes	2.05 (1.43-2.96)	2.00 (1.39-2.90)	NA
Type of hospital control			
Investor owned, for profit	1 [Reference]	1 [Reference]	NA
Nongovernment, not for profit	1.07 (0.86-1.34)	1.08 (0.87-1.35)	NA
Government, nonfederal	1.62 (1.23-2.14)	1.58 (1.19-2.09)	NA
Region			
Other regions	1 [Reference]	1 [Reference]	NA
New England	1.65 (1.12-2.43)	1.69 (1.15-2.49)	NA

Abbreviations: LASSO, least absolute shrinkage and selection operator; NA, not applicable; OR, odds ratio.

When adding an interaction term between safety-net status and teaching intensity, safety-net hospitals with major teaching intensity (OR, 2.15 [95% CI, 1.14-4.03]) were more likely to be penalized than non–safety-net nonteaching hospitals (Table 2). In an alternative model in which teaching hospitals were defined as members of COTH or having major or very major teaching intensity, teaching hospitals remained associated with HACRP penalization (OR, 1.55 [95% CI, 1.15-2.07]). Similarly, when defining safety-net hospitals as hospitals in the highest tertile of the DSHP, safety-net status remained associated with penalization (OR, 1.24 [95% CI, 1.04-1.48]). The variable resident to bed ratio was also included in an alternative model as a continuous variable using restricted cubic splines. The results of that model were consistent with our results that used teaching intensity categorization.

### Association of Hospital Characteristics and HACRP Total Performance Scores

There were 3162 hospitals that had HACRP performance scores available. Although Maryland hospitals did not receive an HACRP penalization status, their HACRP performance scores were available in the CMS databases and therefore were included in this analysis. In the HACRP, higher scores represent worse hospital performance and vice versa. The range of scores in FY 2020 was −1.744 to 2.357. Median (IQR) performance scores were 0.634 (0.450-0.861) for penalized hospitals and −0.202 (−0.531 to 0.044) for nonpenalized hospitals.

Hospital characteristics associated with higher (worse) HACRP scores in the quantile regression model at the 50th quantile (median) included major teaching intensity, safety-net status, and large and medium hospital bed size. In contrast, accreditation by the DNV was associated with lower (better) scores. In the model at the 75th quantile (worst-performing quantile), very major teaching intensity, safety-net status, and non–federal government hospitals were associated with worse scores. Accreditation by the DNV was associated with better HACRP scores. In the model at the 25th quantile (best-performing quantile), larger number of admissions and non–federal government hospitals were associated with worse scores.

The 46 Maryland hospitals assessed had higher (worse) scores than 3116 hospitals located in other states (0.307 [IQR, −0.042 to 0.567] vs −0.040 [IQR, −0.406 to 0.328]; *P* < .001). Compared with hospitals in other states, Maryland hospitals were more likely to be not for profit, have more admissions, and have more accreditations (Joint Commission, DNV, or Commission on Cancer) but had similar proportions of teaching and safety-net institutions.

### Hospital Structure and Reversal of HACRP Penalization

There were 3074 hospitals with penalty status available for FY 2020 and 2021, including 761 of 771 hospitals (98.7%) that had been penalized in FY 2020 (Table 3). Of those penalized hospitals, 268 (35.2%) reverted their penalization status in FY 2021.

**Table 3.** Characteristics of 3074 Hospitals Penalized and Nonpenalized by HACRP in Fiscal Years 2020 and 2021

Characteristic	Hospitals penalized by HACRP in 2020, No. (%)		Hospitals not penalized by HACRP in 2020, No. (%)	
	Penalized in 2021 (remained penalized)	Not penalized in 2021 (penalization reversal)	Penalized in 2021	Not penalized in 2021
No. of hospitals	493	268	267	2046
Resident to bed ratio				
No teaching (0)	252 (51.1)	180 (67.2)	177 (66.3)	1383 (67.6)
Very minor teaching (0.001-0.049)	50 (10.1)	27 (10.1)	18 (6.7)	209 (10.2)
Minor teaching (0.050-0.249)	84 (17.1)	36 (13.4)	46 (17.2)	297 (14.5)
Major teaching (0.250-0.599)	67 (13.6)	17 (6.3)	20 (7.5)	126 (6.2)
Very major teaching (≥0.600)	40 (8.1)	8 (3.0)	6 (2.3)	31 (1.5)
Safety-net hospital				
No	315 (63.9)	194 (72.4)	196 (73.4)	1601 (78.2)
Yes	178 (36.1)	46 (27.6)	71 (26.6)	448 (21.8)
Hospital bed size category				
Small (<100 beds)	120 (24.3)	91 (34.0)	85 (31.8)	735 (35.9)
Medium (100-399 beds)	249 (50.5)	144 (53.7)	149 (55.8)	1058 (51.7)
Large (≥400 beds)	124 (25.2)	33 (12.3)	33 (12.4)	253 (12.4)
Total hospital admissions				
Quartile 1 (≤2507)	86 (17.4)	72 (26.9)	67 (25.1)	544 (26.6)
Quartile 2 (2508-6746)	99 (20.1)	71 (26.4)	73 (27.3)	525 (25.7)
Quartile 3 (6747-13 923)	136 (27.6)	72 (26.9)	70 (26.2)	491 (24.0)
Quartile 4 (>13 923)	172 (34.9)	53 (19.8)	57 (21.4)	486 (23.7)
Case mix index				
Quartile 1 (≤1.41)	99 (20.1)	69 (25.8)	70 (26.2)	531 (26.0)
Quartile 2 (1.42-1.62)	122 (24.7)	67 (25.0)	83 (31.1)	496 (24.2)
Quartile 3 (1.63-1.83)	128 (26.0)	72 (26.9)	56 (21.0)	513 (25.1)
Quartile 4 (>1.83)	144 (29.2)	60 (22.4)	58 (21.7)	506 (24.7)
Accredited by Joint Commission				
No	123 (24.9)	49 (18.3)	60 (22.5)	485 (23.7)
Yes	370 (75.1)	219 (81.7)	207 (77.5)	1561 (76.3)
Accredited by DNV				
No	448 (90.9)	249 (92.9)	249 (93.3)	1821 (89.0)
Yes	45 (9.1)	19 (7.1)	18 (6.7)	225 (11.0)
Commission on Cancer accreditation				
No	248 (50.3)	159 (59.3)	168 (62.9)	1227 (60.0)
Yes	245 (49.7)	109 (40.7)	99 (37.1)	819 (40.0)
Council of Teaching Hospitals				
No	410 (83.2)	245 (91.4)	250 (93.6)	1939 (94.8)
Yes	83 (16.8)	23 (8.6)	17 (6.4)	107 (5.2)
Transplant services				
No	308 (62.5)	179 (66.8)	195 (73.0)	1466 (71.7)
Yes	185 (37.5)	89 (33.2)	72 (27.0)	580 (28.3)
Level I trauma center				
No	399 (80.9)	247 (92.2)	245 (91.8)	1947 (95.2)
Yes	94 (19.1)	21 (7.8)	22 (8.2)	99 (4.8)
Nurse to bed ratio				
Quartile 1 (≤1.09)	123 (25.0)	57 (21.3)	60 (22.5)	529 (25.8)
Quartile 2 (1.10-1.50)	97 (19.7)	74 (27.6)	78 (29.2)	519 (25.4)
Quartile 3 (1.51-1.97)	118 (23.9)	66 (24.6)	66 (24.7)	519 (25.4)
Quartile 4 (>1.97)	155 (31.4)	71 (26.5)	63 (23.6)	479 (23.4)
Inpatient surgeries per bed ratio				
Quartile 1 (≤7.13)	119 (24.1)	71 (26.5)	78 (29.2)	501 (24.5)
Quartile 2 (7.14-10.69)	121 (24.5)	64 (23.9)	59 (22.1)	524 (25.6)
Quartile 3 (10.70-14.70)	131 (26.6)	68 (25.4)	74 (27.7)	496 (24.2)
Quartile 4 (>14.70)	122 (24.8)	65 (24.2)	56 (21.0)	525 (25.7)
Type of hospital control				
Investor owned, for profit	85 (17.2)	62 (23.1)	51 (19.1)	510 (24.9)
Nongovernment, not for profit	302 (61.3)	168 (62.7)	170 (63.7)	1290 (63.1)
Government, nonfederal	106 (21.5)	38 (14.2)	46 (17.2)	246 (12.0)
Hospital region				
New England	30 (6.1)	15 (5.6)	15 (5.6)	70 (3.4)
Other regions	463 (93.9)	253 (94.4)	252 (94.4)	1976 (96.6)

Abbreviations: DNV, Det Norske Veritas; HACRP, Hospital-Acquired Condition Reduction Program.

When compared with hospitals that remained penalized, hospitals that reverted their penalization in 2021 were less likely to be safety-net institutions, be larger, have more admissions, be accredited by the Commission on Cancer, be members of COTH, be level I trauma centers, and be non–federal government hospitals vs for-profit hospitals (Table 3). Hospitals with higher teaching intensity were also less likely to revert their penalization status. For instance, only 16.7% of very major and 20.2% of major teaching hospitals reverted their penalization status compared with 41.7% of nonteaching hospitals (*P* < .001). Hospitals accredited by the Joint Commission were more likely to revert their penalization status (Table 3).

In our multivariable model, safety-net hospitals (OR, 0.64 [95% CI, 0.43-0.96]) and hospitals with higher numbers of admissions (third vs lowest quartile OR, 0.61 [95% CI, 0.37-0.99] and highest vs lowest quartile OR, 0.36 [95% CI, 0.20-0.66]) were less likely to revert penalization. In contrast, hospitals accredited by the Joint Commission (OR, 1.84 [95% CI, 1.24-2.72]) and hospitals with higher nurse to bed ratios (second vs lowest quartile, OR, 1.77 [95% CI, 1.14-2.74]) were more likely to revert their penalization status (Table 4).

**Table 4.** Hospital Characteristics Associated With Hospital-Acquired Condition Reduction Program Penalty Reversal (Categorical Outcome)

Characteristic	Backward elimination model, OR (95% CI)	Model with interaction term, OR (95% CI)	LASSO inference for teaching and safety-net hospitals, OR (95% CI)
Resident to bed ratio			
No teaching	1 [Reference]	1 [Reference]	1 [Reference]
Very minor teaching	1.03 (0.58-1.81)	0.93 (0.49-1.76)	1.00 (0.58-1.71)
Minor teaching	0.89 (0.54-1.49)	0.86 (0.49-1.51)	0.95 (0.59-1.52)
Major teaching	0.65 (0.33-1.35)	0.83 (0.38-1.80)	0.61 (0.31-1.19)
Very major teaching	0.56 (0.23-1.37)	1.22 (0.37-4.03)	0.55 (0.23-1.31)
Safety-net hospital			
No	1 [Reference]	1 [Reference]	1 [Reference]
Yes	0.64 (0.43-0.96)	0.71 (0.41-1.24)	0.63 (0.43-0.93)
Teaching × safety-net interaction			
No teaching, no safety-net	NA	1 [Reference]	NA
Very minor teaching, safety-net	NA	1.43 (0.42-4.90)	NA
Minor teaching, safety-net	NA	1.16 (0.38-3.50)	NA
Major teaching, safety-net	NA	0.49 (0.14-1.80)	NA
Very major teaching, safety-net	NA	0.23 (0.04-1.30)	NA
Accredited by Joint Commission			
No	1 [Reference]	1 [Reference]	NA
Yes	1.84 (1.24-2.72)	1.83 (1.23-2.71)	NA
Total hospital admissions			
Quartile 1	1 [Reference]	1 [Reference]	
Quartile 2	0.85 (0.56-1.31)	0.85 (0.55-1.30)	NA
Quartile 3	0.61 (0.37-0.99)	0.61 (0.37-1.00)	NA
Quartile 4	0.36 (0.20-0.66)	0.36 (0.86-2.20)	NA
Nurse to bed ratio			
Quartile 1	1 [Reference]	1 [Reference]	
Quartile 2	1.77 (1.14-2.74)	1.77 (1.14-2.75)	NA
Quartile 3	1.39 (0.88-2.18)	1.40 (0.89-2.21)	NA
Quartile 4	1.39 (0.87-2.22)	1.37 (0.86-2.20)	NA

Abbreviations: LASSO, least absolute shrinkage and selection operator; NA, not applicable; OR, odds ratio.

## Discussion

Our cross-sectional study found that teaching and safety-net hospitals continued to be associated with disproportionate penalization by the HACRP despite recent changes in the program’s methodology. As in previous studies,^17,18,27^ we observed a stepladder effect in penalization status as hospital teaching intensity increased. For instance, one-third of major teaching hospitals and half of very major teaching hospitals were penalized compared with less than a quarter of nonteaching hospitals. This overpenalization does not appear to be supported by other studies comparing teaching and nonteaching hospitals that have demonstrated similar performance on clinical outcomes, such as readmission and mortality rates.^27,28^

Consistent with previous studies,^17,27,29^ safety-net hospitals had a 41% increase in the risk of penalization compared with non–safety-net hospitals. The interaction between safety-net and teaching intensity resulted in far worse outcomes for these hospitals. Safety-net hospitals with major teaching levels were twice as likely to be penalized as non–safety-net nonteaching hospitals.

A hypothesis for the persistent overpenalization of teaching and safety-net hospitals arises from the lack of inclusion of risk adjustment for complex socioeconomic determinants of care in HACRP.^13,17,18,19^ A clear example of the potential benefit of controlling for complex socioeconomic factors was observed in the Hospital Readmission Reduction Program, in which the stratification of hospitals based on their proportion of patients dually enrolled in Medicare and Medicaid (who have a significant number of health comorbidities and social determinants of care) decreased the overpenalization of teaching and safety-net hospitals.^30^ The results of a recent study^31^ of hospitals participating in HACRP FY 2020 suggested that if stratification based on the proportion of dually enrolled patients had been used, this may have resulted in a significant reduction of penalties for safety-net hospitals.

Hospitals in the New England region were also found to be more likely to be penalized than those in other regions. A previous study^18^ reported that HACRP penalization rates in New England hospitals had increased over the years, reaching 27% in 2015 and 39% in 2017. Those penalization rates were higher than rates in other regions. Although New England had a higher percentage of teaching hospitals than other regions (48% vs 34%, *P* < .001), the definitive reasons for the overpenalization of this region need to be further explored.

As in earlier studies,^17,18,19,27^ larger hospitals offering more specialized services (including transplant, cancer, and advanced trauma services) were more frequently penalized by HACRP. In contrast to a prior study,^17^ hospitals accredited by the Joint Commission or DNV were not associated with HACRP penalization. Indeed, accreditation by the DNV was associated with better performance scores in HACRP FY 2020.

We also evaluated hospital characteristics associated with the reversal of HACRP penalization and found that safety-net hospitals and hospitals with higher numbers of admissions were less likely to revert their penalization status. Among teaching hospitals that were penalized in FY 2020, less than 20% of major and very major teaching hospitals reverted their penalization status in FY 2021 compared with more than 40% of nonteaching hospitals. These results are similar to a previous study,^18^ in which larger teaching institutions were less likely to improve their HACRP performance scores over a 3-year period (2015-2017) than smaller nonteaching hospitals. In our study, hospitals accredited by the Joint Commission were more likely to revert their penalization status. The Joint Commission has been actively involved in developing evidence-based and actionable prevention measures to reduce the number of health care infections.^32^ Whether the more stringent implementation of their recommendations by penalized hospitals contributed to their penalty reversal deserves further study.

The HACRP was designed to penalize the lowest quartile of performing hospitals every year despite potential improvements in their performance scores.^18^ Rather than leading to future improvement, penalization of safety-net and teaching hospitals seems destined to perpetuate disparities. Financial penalties levied by the HACRP may cause further financial constraints on teaching and safety-net institutions, impairing the quality of care they offer to vulnerable underserved populations.^10,17^

### Strengths and Limitations

One of the main advantages of our study is the inclusion of all acute care hospitals participating in the HACRP, which enhanced the internal and external validity of our results. This study has limitations. Due to the COVID-19 pandemic, the CMS exempted hospitals from the mandatory reporting of HAI data for the last quarter of 2019. This may have impaired the determination of HACRP penalties in FY 2021 and affected our analysis of penalty reversal associations. As with any other project using secondary data, limitations related to incomplete or inconsistent data reporting are also possible.

## Conclusions

Findings from this cross-sectional study suggested an ongoing overpenalization of teaching and safety-net hospitals. The overpenalization of these hospitals and a decreased probability of penalty recovery may exhaust resources available to care for underserved populations. It is urgent that measures be rapidly adopted to avoid widening disparities.

## Notes

Supplement 1. **eFigure.** Timeline of reported hospitalizations in different databases

Supplement 2.
Data Sharing Statement
