# Supplementary material for: Teaching and Safety-Net Hospital Penalization in the Hospital-Acquired Condition Reduction Program
Source: JAMA Netw Open. 2024 Feb 16;7(2):e2356196. doi: 10.1001/jamanetworkopen.2023.56196 (PMC10873765; doi:10.1001/jamanetworkopen.2023.56196)
Supplement: Supplement 1. — eFigure. Timeline of reported hospitalizations in different databases [file jamanetwopen-e2356196-s001.pdf]

Supplementary Online Content

Serpa JA, Gemeinhardt G, Arias CA, et al. Teaching and safety-net hospital penalization in the Hospital-Acquired Condition Reduction Program. *JAMA Netw Open*. 2024;7(2):e223050. Doi:10.1001/jamanetworkopen.2023.56196

eFigure. Timeline of reported hospitalizations in different databases

This supplementary material has been provided by the authors to give readers additional information about their work.

© 2024 Serpa JA et al. *JAMA Network Open*.

eFigure. Timeline of reported hospitalizations in different databases

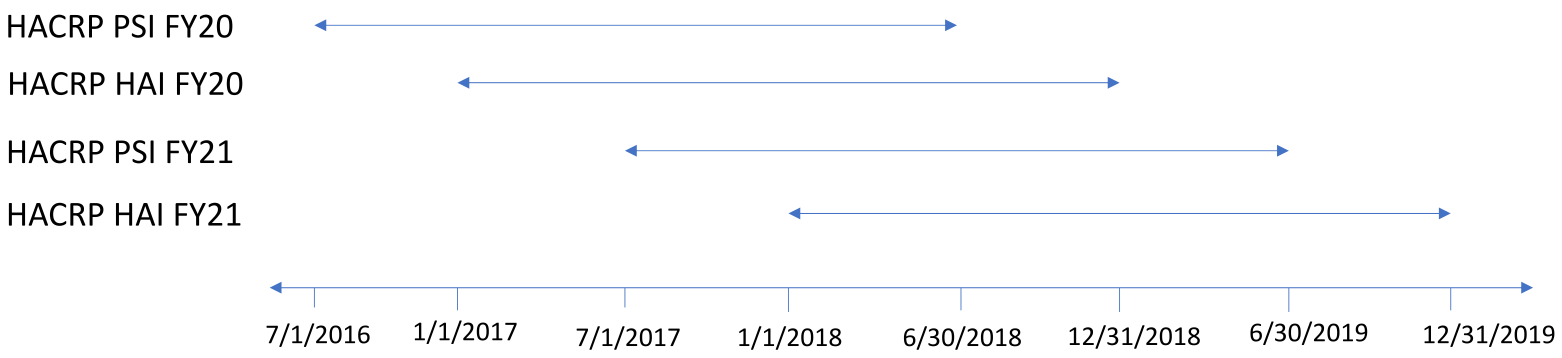

HACRP: Hospital acquired condition reduction program
PSI: Patient safety indicator
HAI: Healthcare associated infection
FY: Fiscal year

© 2024 Serpa JA et al. *JAMA Network Open*.
