# Supplementary material for: Teaching and Safety-Net Hospital Penalization in the Hospital-Acquired Condition Reduction Program
Source: JAMA Netw Open. 2024 Feb 16;7(2):e2356196. doi: 10.1001/jamanetworkopen.2023.56196 (PMC10873765; doi:10.1001/jamanetworkopen.2023.56196)
Supplement: Supplement 2. — Data Sharing Statement [file jamanetwopen-e2356196-s002.pdf]

Data Sharing Statement

Serpa. Teaching and Safety-Net Hospital Penalization in the Hospital-Acquired Condition Reduction Program. *JAMA Netw Open*. Published February 16, 2024. doi:10.1001/jamanetworkopen.2023.56196

Data
Data available: No

Additional Information
Explanation for why data not available: Data used in this study is publicly available
